# Quantifying the Impact of Land Cover Composition on Intra-Urban Air Temperature Variations at a Mid-Latitude City

**DOI:** 10.1371/journal.pone.0102124

**Published:** 2014-07-10

**Authors:** Hai Yan, Shuxin Fan, Chenxiao Guo, Jie Hu, Li Dong

**Affiliations:** 1 College of Landscape Architecture, Beijing Forestry University, Beijing, China; 2 School of Landscape Architecture, Zhejiang Agriculture and Forestry University, Lin’an, Zhejiang, China; 3 School of architecture, Tsinghua University, Beijing, China; 4 Research Center for landscape architecture, Beijing Tsinghua Urban Planning & Design Institute, Beijing, China; University of Kent, United Kingdom

## Abstract

The effects of land cover on urban-rural and intra-urban temperature differences have been extensively documented. However, few studies have quantitatively related air temperature to land cover composition at a local scale which may be useful to guide landscape planning and design. In this study, the quantitative relationships between air temperature and land cover composition at a neighborhood scale in Beijing were investigated through a field measurement campaign and statistical analysis. The results showed that the air temperature had a significant positive correlation with the coverage of man-made surfaces, but the degree of correlation varied among different times and seasons. The different land cover types had different effects on air temperature, and also had very different spatial extent dependence: with increasing buffer zone size (from 20 to 300 m in radius), the correlation coefficient of different land cover types varied differently, and their relative impacts also varied among different times and seasons. At noon in summer, ∼37% of the variations in temperature were explained by the percentage tree cover, while ∼87% of the variations in temperature were explained by the percentage of building area and the percentage tree cover on summer night. The results emphasize the key role of tree cover in attenuating urban air temperature during daytime and nighttime in summer, further highlighting that increasing vegetation cover could be one effective way to ameliorate the urban thermal environment.

## Introduction

Humanity is rapidly urbanizing. Whereas in 1950 a mere 29% of the world population lived in urban areas, this proportion now exceeds 52% and is expected to reach 67% by 2050, and much of this growth occurring in developing nations such as China and India [1]. Growing urban populations and urban expansion, with increasing built-up areas and human activities, results in significant modifications in the underlying surface properties and energy balance, and thus alters the urban climate [2]–[4]. The most well-documented example of urban climate modification is the urban heat island (UHI) effect, which refers to the phenomenon of higher air and surface temperatures occurring in urban areas than in the surrounding rural areas [5], [6].

Urban climate is influenced by many factors related to the intrinsic nature of a city. One of the most important causal factors is the conversion of land cover from rural to urban covers, mainly the replacement of natural vegetation covers by impervious man-made surfaces [3]. The composition and arrangement of natural and man-made surfaces, including impervious surfaces, vegetation areas and water body, as well as the local weather conditions influence near surface energy flux partitioning, resulting in variable local climates [5]. For example, vegetation cover and composition has been shown to be important for explaining spatial difference in urban and suburban air temperatures [7]–[10]. Some studies have also used remote sensing technology to evaluate land surface temperature distribution patterns and estimate the UHI for a number of different urban localities, especially at vast geographic scales [11], [12]. Using a variety of image data, these studies found that land cover characteristics, such as vegetation indices measured by NDVI (normalized difference vegetation index), and the relative amounts of other land cover types (building area, impervious surface, water body) significantly affect the urban temperature distribution patterns and UHI intensity [13]–[20].

Although the urban area was generally warmer than the surrounding suburban and rural areas, the temperature distribution is not a simple urban-rural gradient [16]. On a smaller scale in the city, there are also surprisingly large temperature differences [7]. Urban areas with varied land cover often comprise a mosaic of warm and cold areas that vary significantly within the micro to local scale (10 m to 10 km) [21], [22]. Air temperatures at different points within the same urban area may differ significantly even in the same overall climatic context, and they can be affected by the thermal state of the adjacent surface cover, and are also affected by dispersion through turbulence and advection from surroundings. For example, many field-based measurements have found that urban green areas such as parks are generally cooler than their surrounding built-up areas, and can produce air temperature differences up to 1–7°C, a phenomenon sometimes referred to as a “park cool island” [23]–[30]. However, the actual extent of this cooling may vary significantly, depending on the characteristics of the park, such as park size and park cover composition [27], [31]. Variation in the composition of surface covers within a park, such as the amount of trees, turfs and paved surfaces can be expected to affect temperature. A small number of studies also showed that the cooling effect of vegetation could extends into its surrounding area [32]–[34], which will contributes to ameliorating outdoor thermal environment and mitigating the UHI effect. Furthermore, a few quantitative associations between air temperature and its surrounding land cover pattern have been reported [35]–[38]. Krüger and Givoni [35], for example, suggested a relationship between seven air temperature variations and land cover pattern around each location in Curitiba, Brazil. Sun [36] showed air temperature significantly correlated with green ratio and building ratio at night in Taichung City, Taiwan. Yokobori and Ohta [37] investigated the effect of land cover on ambient air temperatures in a suburb of the Tokyo metropolitan area, Japan, and found that air temperatures varied significantly according to ambient land cover types, and air temperatures decreased as the amount of vegetated area around the sites increased.

As this brief overview shows, the effects of land cover, especially vegetation, on urban temperature are well documented. Most of these studies are implemented at the scale of an entire city, but little information is available for the microclimatic conditions at urban design scale. In cities, however, planning and design strategies aimed at the avoidance of additional heat loads are generally applied at the scale of a city block, a neighborhood, or an individual building. This deficiency makes it difficult to address satisfactorily a growing demand in urban climate knowledge for solutions to practical questions in urban design process to create more comfortable and healthy communities. In addition, a comprehensive understanding of how much influence the different land cover types have on the urban air temperature variations and how the heterogeneity of land covers affects temperature variations on multiple temporal and spatial scales is still lacking. Furthermore, understanding the exact relationship between air temperature and land cover should isolate the urban warming effects from other warming influences such as urban morphology, topography, and weather conditions.

Given the above issues, this study investigates the quantitative relationships between air temperature and the composition of land cover features at a local scale in Beijing, China. Specifically, we aim to elucidate: 1) To what extent does the conversion of land cover from rural natural surfaces to man-made surfaces affect the urban air temperature variations, and the seasonal and diurnal variation of the relationship; 2) what spatial extent is air temperature at a point affected by the composition of land cover conditions around that location; and 3) if the relative importance of different land cover types in explaining air temperature difference varies with time and extent expansion.

## Materials and Methods

### Ethics statement

The field studies were conducted at an urban public open space in Beijing city. Thus, we could conduct experiments there without specific permits. The experiments conducted in this study did not involve endangered or protected species.

### Study area

Beijing city (39°56′N, 116°20′E), the capital of China, is located in the northern part of the North China Plain. It is the second largest city in China with a total population of 19.6 million by the end of 2010. It has a monsoon influenced humid continental climate characterized by hot and humid summers and generally cold, windy and dry winters. January is the coldest month with mean air temperature below –3.7°C, while July is the hottest month with mean air temperature over 26.2°C. The annual average temperature is 12.3°C, with an average precipitation of 630 mm/year. The main wind direction is from southeast to northeast in summer and in reverse during winter. Since 1978, Beijing’s urban population and yearly construction area have been gradually increasing. This urbanization process, with its increasing built-up areas and anthropogenic activities, results in significant modifications in the underlying surface properties and the increase in the intensification of the UHI effect [39].

**Figure 1:**
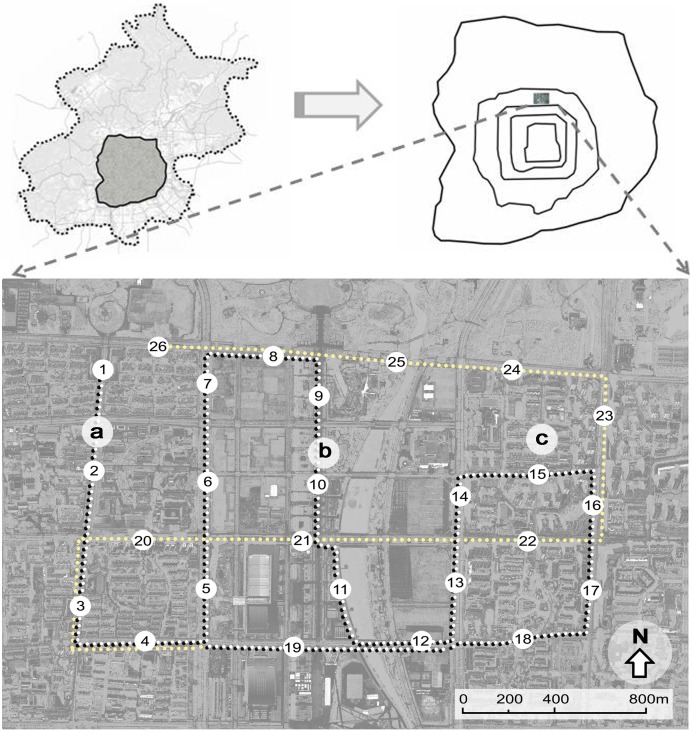
A map of the study area and location of measurement points. The numbers represent the mobile measurement points, while the lowercase letters represent the three fixed air temperature measurement sites.

**Figure 2:**
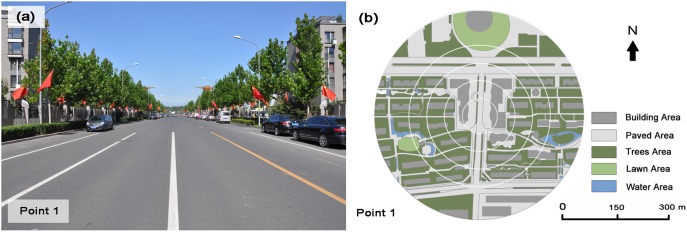
Photographs (a) and land cover classification (b) of one example of the measurement point (points 1). The circles represent the different spatial extent scale from 20 to 300

The measurements took place at the Beijing Olympic Park and its surrounding built-up areas in Beijing city (Figure 1). The study area is rectangular, 3.2×2.1 km in size and situated at the northern part of the city. It can be regarded as a typical area in the process of urbanization in Beijing. This site was chosen because 1) it is a very flat area with an average altitude of 50 m, therefore temperature difference due to topographic influence can be ignored; 2) this district has a perfectly orthogonal urban geometry and the streets are oriented E–W and N–S, with an average width of 25 m; 3) it is comprised of various land cover types including building area, paved road, vegetation, and water area; 4) The overall climatic context is mostly constant across the study area. Therefore, it is a good study area to explore the relationship between air temperature and land cover pattern. In this study area, 26 measurement points were chosen with the intention to investigate the quantitative relationship between air temperature and the composition of land cover features (Figure 1). Since the observation sites were limited to several square kilometers, they could be affected by uniform meso-scale urban canopy layer climate condition. However, each measurement point was located at intervals of approximately 300 to 600 m, so they were also affected by distinctly different micro-scale factors. The nature of the adjacent underlying surface greatly affects air temperature. Thus, to standardize temperature, measurements were made over a common surface: paved road, as shown in Figure 2a.

### Air temperature measurements

Spatial variations in canopy layer temperature can be observed via mobile traverses, which can accurately depict the spatial distribution of microclimate over the target areas for focused surveys upon small time and space scales. In the present study, mobile traverses were used to conduct air temperature measurements during 4 winter days (December 2012 to February 2013) and 4 summer days (June to August 2013). Survey times for each day were early afternoon (14∶00–15∶00) and midnight (23∶00–00∶00). All observations were carried out during clear and windless days, i.e. when the UHI effects could be expected to be most pronounced. Avoidance of windy conditions also minimizes the influence that the air temperature of a measurement might have on surrounding sites through convection.

Measurements of air temperature were taken using a mobile station with a thermistor temperature sensor connected to a data logger with a precision of 0.1°C along the traverse route within the study area. The temperature sensor was fitted within a radiation shield, which mounted onto the front of a bicycle at 1.5 m above ground level. A GPS travel recorder was used to note geographical coordinates of latitude, longitude, and altitude synchronized to observations. Three fixed weather stations were installed in a different region within the study area for recording of the daily trend of air temperature. These data were used to correct the values taken with the mobile station because the measurements at different sites were not instantaneous. Each traverse took about 1-hour to complete, and all data were adjusted to the beginning time of each transect by modifying values using temperature change rates over the study area based on fixed weather station measurements of air temperature.

### Land cover classification

Satellite imagery (Google Earth satellite image, September 15, 2012) was used to classify the land cover types in the study area. Land cover types were firstly classified into two broad categories: man-made surfaces and natural surfaces on the basis of the permeability and thermal properties, which represent urban surface and rural surface, respectively. Then the man-made surfaces were subclassified into building area and paved area, and natural surfaces were subsequently divided into tree area, lawn area and water area.

The qualitative land cover conditions must be quantified to be used in correlation analyses. A buffer zone was used to accomplish this. As previous studies showed that a very strong correlation exists between air temperature at a point and land cover composition within a certain area of that location [35]–[38]. However, the extent of buffer zone varied greatly in different studies. Sun [36] used buffers as small as 10-m in radius in an urban thermal environment study in Taichung City, while buffers up to 125-m and 300-m in radius have been used, e.g. by Krüger and Givoni [35] in Curitiba, and by Yokobori and Ohta [37] in a suburb of the Tokyo metropolitan area, respectively. In this study, to quantify the effect of spatial extent on relationships between air temperature and land cover pattern, land cover percentage was calculated for each measurement point at 6 levels of spatial extent scale with size of 1257, 7854, 31416, 70686, 125664, and 282743 m^2^ (corresponding to 20, 50, 100, 150, 200, and 300 m in radius around each point, respectively), as shown in Figure 2b. Using a similar method to Krüger and Givoni [35], the percentage of five categories of land cover for each measurement point, considering the different spatial extent scales, were calculated with AutoCAD, by drawing corresponding areas on the aerial photograph.

### Statistical analyses

For each spatial scale, we first performed simple linear regression to analyze the bivariate relationship between air temperatures and the percentage of man-made surfaces. Next, a Pearson correlation matrix was developed to examine the strength of bivariate associations between air temperature and different land cover types. Finally, we further used a multiple regression analysis to examine the relationships between air temperatures and land cover patterns, with an aim to find out how well the spatial temperature differences can be explained by the combination of the percentage of five land cover types at different time and spatial extent scales. All statistical analyses were performed using SPSS 17.0 software.

## Results

### Effects of man-made surfaces on urban air temperature

**Figure 3:**
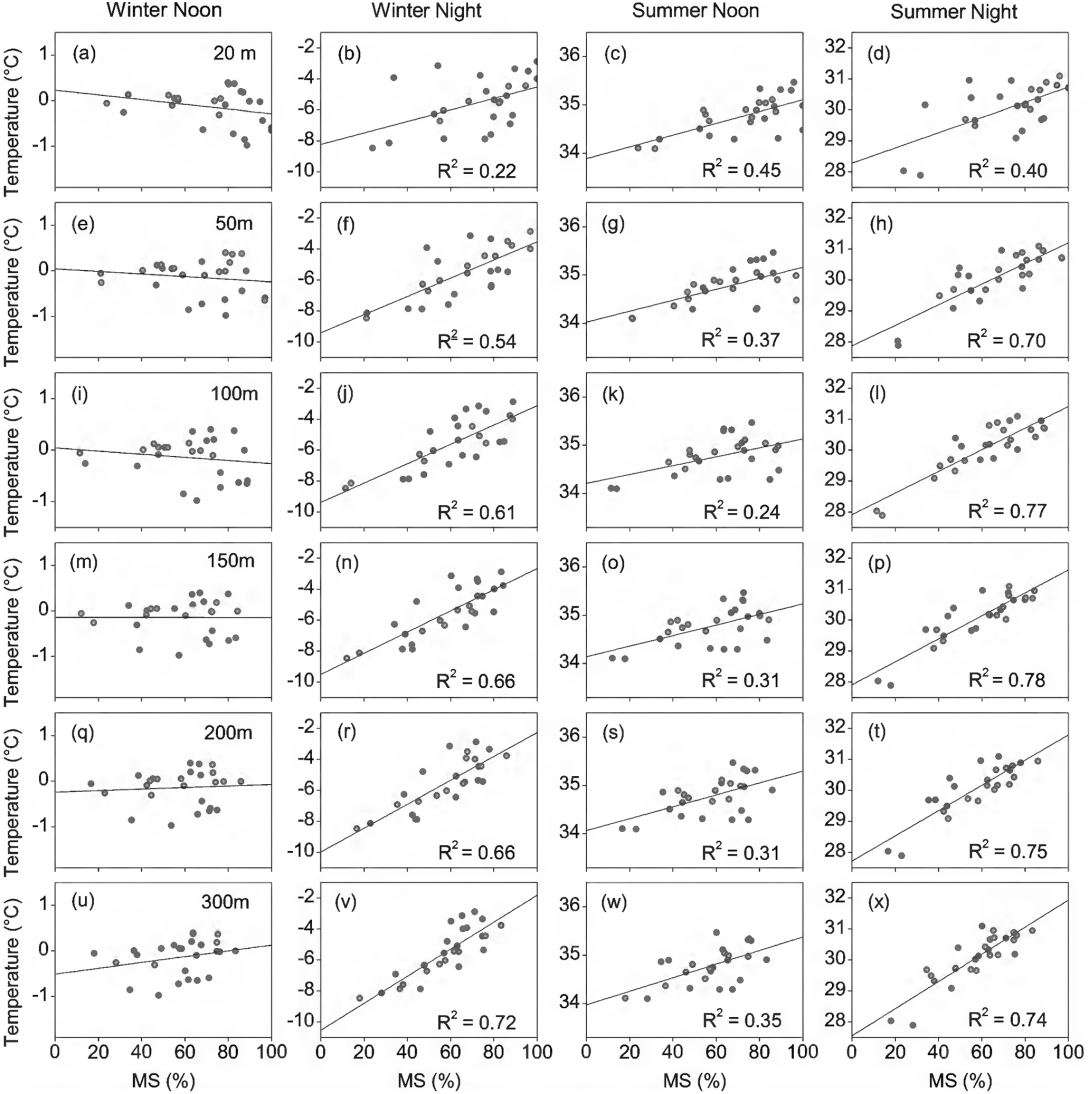
Relationships between air temperature and the percentage of man-made surfaces at different spatial scales. Each row represents the buffer zone with size given by the values on the first column, and the buffer size increases from top to bottom. From left to right, the four columns show the patterns of winter noon, winter night, summer noon and summer night of temperature– manmade surfaces relationship, respectively.

Figure 3 shows the relationships between air temperatures and the percentage of man-made surfaces (PerMS) at different temporal and spatial scales. At noon in winter, there was a fairly weak correlation between air temperatures and the PerMS. It can be seen that the slope transitions were from negative (at scale<150 m), relatively flat (at scale of 150 m), to positive (at scale>150 m) slopes. However, for other three time periods, air temperature shown a significant and positive correlation with the PerMS. Thus, the higher the PerMS in an urban context the greater the air temperature is to be expected in the area. In general, the magnitude of the air temperature variation of the four time periods complied with the order at different scales: winter night > summer night > summer noon > winter noon. The maximum air temperature variations (MaxATV), which was due to the conversion of land cover from rural natural surfaces (0% of MS) to almost complete man-made surfaces (100% of MS), were 3.7–8.7°C, 2.5–4.5°C, 0.9–1.4°C, and –0.3–0.6°C for winter night, summer night, summer noon and winter noon, respectively.

### Effects of spatial scale on relationship between temperature and man-made surfaces

**Figure 4:**
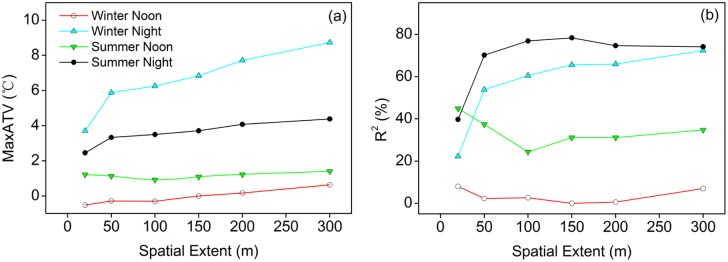
Changes in the maximum air temperature variation (MaxATV) values (a) and explanatory power, R^2^ values (b) of regression models for air temperatures as a function of the percentage of man-made surfaces with spatial scale during daytime and nighttime in winter and summer.

It shows clearly that spatial scale (the buffer zone size) significantly influenced the relationship between air temperatures and the PerMS. As quantified by the MaxATVs of these relationships, the magnitude of MaxATVs increased consistently with the increasing spatial scale, as shown in Figure 4a. The increasing trends were especially obvious during nighttime, especially during the winter night. Moreover, spatial extent scale substantially affected the explanatory power (R^2^) of the PerMS on air temperatures (Figure 4b). On summer nights, the R^2^ rapidly increased from 39.7% (20 m), to 70.2% (50 m), 76.9% (100 m), and peaked at the scale level of 150 m (78.4%), and then slowly decreased to 74.7% (200 m), 74.1% (300 m). Similarly, on winter nights, the R^2^ rapidly increased with increasing buffer zone size at spatial scale <150 m, maintaining a constant within the wider areas. In contrast, at noon in summer, the R^2^ decreased from 44.9% (20 m) to about 24.4% (100 m), and increased to 31.1% (150 m), and then remained at about 31–34% with a further increase in spatial scale. At noon in winter, there was little or no variation in the explanatory power of the PerMS on air temperatures, which maintained at a low level about 0–8% at different spatial scales.

### Effects of land cover type on air temperature

The correlation coefficients indicate that the relationships between air temperatures and land cover types at various spatial extent scales differed among the four time periods (Table 1). For winter noon, there was not a significant relationship between air temperatures and any of the land cover types. On winter nights, all of the land cover variables, except the percentage of water area, were significantly related to air temperatures. Air temperature had a positive relationship with the percentage of building area (PerBA) and the percentage of paved area (PerPA), but a negative relationship with the percentage tree cover (PerTC) and the percentage of lawn area (PerLA). The greatest correlation between air temperature and the PerBA, PerPA, PerTC, and PerLA occurred at the spatial scales of 200 m, 100 m, 150 m and 300 m in radius, respectively. At noon in summer, there was a significant positive relationship between air temperatures and the PerBA and PerPA, and a negative relationship between air temperatures and the PerTC. The degree of correlation between air temperature and the PerBA increased with increasing spatial scale, and peaked at the spatial extent of 300 m in radius, while for the PerPA and PerTC the reverse trend was observed. On summer nights, the pattern of relationships between air temperature and the five land cover types were very similar to the pattern of relationships on winter nights. However, the PerTC had a stronger negative relationship with air temperature than the PerLA during summer nights.

**Table 1:** Correlation coefficients between air temperature and land cover types at different temporal and spatial scales.

Scale	20 m	50 m	100 m	150 m	200 m	300 m
*Winter noon*						
PerBA	−0.073	−0.155	−0.021	0.017	0.023	0.120
PerPA	−0.305	−0.093	−0.229	−0.021	0.095	0.293
PerTC	0.210	−0.079	−0.102	−0.216	−0.327	−0.473
PerLA	0.198	0.259	0.258	0.178	0.185	0.129
PerWA	0.103	0.115	0.117	0.120	0.078	−0.026
*Winter night*						
PerBA	0.512**	0.677**	0.745**	0.815**	**0.870** **	0.841**
PerPA	0.295	0.489*	**0.548** **	0.515**	0.481*	0.446*
PerTC	−0.228	−0.474*	−0.485*	−**0.551** **	−0.503**	−0.426*
PerLA	−0.546**	−0.490*	−0.487*	−0.514**	−0.581**	−**0.704** **
PerWA	0.090	−0.024	−0.096	−0.080	−0.156	−0.219
*Summer noon*						
PerBA	0.432*	0.405*	0.439*	0.507**	0.507**	**0.595** **
PerPA	**0.577** **	0.551**	0.378	0.404*	0.412*	0.299
PerTC	−**0.604** **	−0.447*	−0.238	−0.337	−0.401*	−0.458*
PerLA	−0.277	−0.318	−0.319	−0.340	−0.325	−0.337
PerWA	−0.014	−0.122	−0.186	−0.169	−0.149	−0.107
*Summer night*						
PerBA	0.431*	0.642**	0.758**	0.821**	**0.853** **	0.775**
PerPA	0.529**	0.674**	**0.685** **	0.624**	0.575**	0.535**
PerTC	−0.440*	−0.633**	−0.624**	−**0.671** **	−0.664**	−0.565**
PerLA	−0.488*	−0.419*	−0.410*	−0.418*	−0.442*	−**0.537** **
PerWA	0.015	−0.140	−0.233	−0.212	−0.262	−0.285

Note: PerBA, PerPA, PerTC, PerLA and PerWA refer to the percentage of building area, paved area, tree area, lawn area, and water body area respectively. The bold numbers represent the highest correlation coefficient for each land cover type among different spatial extents (from 20 to 300 m in radius).

*Correlation is significant at the 0.05 level (two-tailed).

**Correlation is significant at the 0.01 level (two-tailed).

### Multiple regression analysis

Stepwise multiple regression analysis was carried out in order to find out how well the observed temperature differences can be explained by the combination of different land cover types (Table 2). The R^2^ represent the proportion of the variation in temperature explained by the regression models, and the standardized coefficients of predictive models represent the relative contributions of different land cover types to the air temperature. The results indicate that during the four observation period, it is the summer nighttime air temperatures that can be best explained by four variables, followed by winter nighttime and summer daytime by three variables. Our model specification for winter noon failed to generate any significant results.

**Table 2:** Regression results with the land cover composition as predictor variables and the air temperature as response variables on winter night.

Scale	Untandardized coefficients				Standardized coefficients				R^2^	Adjusted R^2^
	PerBA	PerPA	PerTC	PerLA	PerBA	PerPA	PerTC	PerLA		
20 m				−0.086				−0.546	0.298	0.269
50 m	0.080	0.042			0.595	0.349			0.574	0.537
100 m	0.091	0.040			0.631	0.334			0.653	0.623
150 m	0.104		−0.034		0.710		−0.313		0.751	0.729
200 m	0.132	0.032			0.798	0.218			**0.799**	**0.782**
300 m	0.118	0.051			0.787	0.308			0.799	0.781

Note: PerBA, PerPA, PerTC and PerLA refer to the percentage of building area, paved area, tree area and lawn area, respectively. The bold numbers represent the highest explanatory power of the regression model among different spatial scales (from 20 to 300 m in radius). The stepping method criterion included a variable if it was statistically significant at p = 0.05 level.

On winter nights (Table 2), the R^2^ values vary from 0.298 to 0.799. The highest R^2^ value of 0.799 occurred at the spatial scale of 200 m, indicating that a combination of the PerBA and the PerPA explained 79.9% of the variation in winter night air temperatures. Both of these variables were positively correlated with air temperature. The comparison of standardized coefficients indicated that the PerBA was the most important variable. A 10% increase in building cover resulted in an increase in air temperature of 1.32°C.

At noon in summer (Table 3), the R^2^ values vary from 0.192 to 0.365. The highest R^2^ value of 0.365 occurred at the spatial scale of 20 m, indicating that the predictor accounted for 36.5% of the variation in air temperatures. The PerTC was the only variables selected in this model. A 10% increased in tree cover resulted in a decrease in temperature of 0.12°C.

**Table 3:** Regression results with the land cover composition as predictor variables and the air temperature as response variables at summer noon.

	Untandardized coefficients			Standardized coefficients			R^2^	Adjusted R^2^
Scale	PerBA	PerPA	PerTC	PerBA	PerPA	PerTC		
20 m			−0.012			−0.604	**0.365**	**0.339**
50 m		0.015			0.551		0.304	0.275
100 m	0.015			0.439			0.192	0.159
150 m	0.017			0.507			0.257	0.226
200 m	0.019			0.507			0.258	0.227
300 m	0.021			0.595			0.353	0.326

On summer nights (Table 4), the R^2^ values vary from 0.406 to 0.871. The highest R^2^ value of 0.871 occurred at the spatial scale of 200 m, which indicating that a combination of the PerBA and the PerTC explained 87.1% of the variation in air temperatures. The PerBA was positively correlated with air temperature, while the PerTC had a negative correlation. A 10% increase in building cover increased temperature by 0.58°C, whereas a 10% increase in tree cover decreased temperature by 0.27°C.

**Table 4:** Regression results with the land cover composition as predictor variables and the air temperature as response variables on summer night.

	Untandardized coefficients			Standardized coefficients			R^2^	Adjusted R^2^
Scale	PerBA	PerPA	PerTC	PerBA	PerPA	PerTC		
20 m	0.032	0.022		0.359	0.474		0.406	0.354
50 m	0.034	0.033		0.512	0.554		0.702	0.676
100 m	0.043	0.029		0.595	0.484		0.783	0.764
150 m	0.049		−0.024	0.671		−0.445	0.850	0.836
200 m	0.058		−0.027	0.704		−0.408	**0.871**	**0.860**
300 m	0.052	0.034		0.703	0.411		0.764	0.744

## Discussion

In its essence, urban climate is directly related to the conversion of land cover from rural natural surfaces to urban man-made surfaces. Specifically, the urban air temperature is influenced by the relative proportion of man-made surfaces to natural surfaces. In the present study, we found that air temperature had a significant and positive relationship with the PerMS. Thus, the higher the PerMS in an urban texture the greater the air temperature is to be expected in the area. This result, although conducted at a fine-scale, was consistent with the previous studies which have used different techniques at larger scales [15]. However, our results indicated that the relative impacts of man-made surfaces on air temperature varied among different times and seasons. In general, the magnitude of the impact of man-made surface on air temperature complied with the order during different periods: winter night > summer night > summer noon > winter noon. During the winter, the vegetation withered, and the bright, dry grounds increase in albedo at the same time as lower soil moisture leads to low thermal heat capacity. Less heat is absorbed by the ground at daytime and is more easily lost due to effective radiative cooling during nighttime, and thus the air temperature difference between vegetated area and man-made surface is minor at winter noon and bigger at winter night. Compare to the winter, the vegetation flourishes in summer and the substrate of vegetated area contains more water, therefore, the capacity to store heat also increases. At noon in summer, dense tree canopies function as shade-providers resulting in a decrease in ambient air temperature. On night, tree canopies, however, will reduce the radiative cooling and might act to decrease the magnitude of the nocturnal cooling effect. Thus, the air temperature difference is stronger at summer noon than at winter noon, while the air temperature difference is weaker on summer night compared to winter night.

The effects of land cover types on ambient air temperatures have been extensively documented [35], [37]. Our results indicate that the air temperature increased as the PerBA and PerPA increased. This may be because impervious building and paved surfaces limit the presence of moisture which plays a key role in moderating local air temperatures. In contrast, the air temperature decreased as the PerTC and PerLA increased. This is consistent with those from previous research that vegetation can reduce air temperature through the combined effect of direct shading and evapotranspiration during daytime [23]–[26], [33]. Although the vegetation does not provide shading and evapotranspiration during the night, the uses of the trees and lawn were still cooler than other kind of man-made surfaces. In winter, both of the trees and lawn are withered, and they had rather similar cooling effect. In summer, however, the tree cover had stronger cooling effects. This result emphasizes that in places with hot summer, planting more trees in urban area will help to reduce the air temperature so that to provide a pleasant environment and reduce energy consumption. It was also shown that the relationship between air temperature and the percentage of water area was very poor, the possible reason might be the comparatively few measurement points containing water bodies.

Spatial extent significantly influenced the relationship between air temperatures and land cover. The magnitude of the air temperature variation, which was due to the conversion of land cover from rural natural surfaces to man-made surfaces, increased consistently with increasing spatial extent (from 20 to 300 m in radius). The results also indicated that spatial extent scale substantially affected the explanatory power of the PerMS on air temperature. These findings suggest that the air temperature is affected by a certain spatial extent surrounding it. Moreover, different land cover types also had very different spatial extent dependence: with spatial extent expansion (from 20 to 300 m in radius), the correlation coefficient of different land cover types varied differently, and more interestingly, their relative impacts also varied among different times and seasons. At noon in summer, the highest correlation coefficient between air temperature and the PerTC occurred at the smallest spatial extent of 20 m in radius, which provided evidence of the influence of tree canopy shading on daytime air temperature. This is also consistent with previous studies that have discussed the importance of urban canopy structure (i.e. sky view factor or height/width ratio) as a major driver of spatial patterns of air temperature during daytime [40], [41]. However, on summer night, the highest correlation coefficient between air temperature and the PerTC occurred at the spatial extent of 150 m in radius. This result quantitatively confirmed previous studies that the cooling effect of green areas could extend for several hundred meters [30]. In suburban Tokyo, for example, Yokohari et al. [42] found that the cooling effect of paddy field reaches a steady temperature at approximately 150 m into the neighborhood during summertime.

The results of the multiple regression models indicate that the PerBA and PerPA could best explain variation in air temperature for winter night. Especially, the PerBA was the most important variable on winter night, as a rule of thumb, a 1.32°C increase in ambient air temperature is to be expected for a 10% increase to the PerBA. This result indicates that a more densely clustered arrangement of the buildings is favorable for thermal environment and energy conservation during winter night. At noon in summer, air temperature could best be explained by the PerTC at the spatial extent of 20 m in radius. At this time, the significant negative correlation between urban tree cover and ambient air temperature suggests that the trees shading plays a major role in determining the cooling effect of the site during the day. This is consistent with the investigations of the cooling effect of small vegetated areas by Shashua-Bar and Hoffman [33]. Additionally, we found that air temperature on summer night was better predicted by the PerBA and PerTC. Therefore, during nighttime in summer, increasing percent cover of building could significantly increase air temperature, while the increase of tree cover would significantly decrease air temperature, and thus help to mitigate excess heat in urban areas. These findings emphasizes the key role of tree cover in mitigating urban air temperature during hot summer night, further highlights that increasing urban vegetation cover could be one of effective way to ameliorate the urban thermal environment.

## Conclusions

The relationships between urban air temperature and the composition of land cover conditions at a neighborhood scale in Beijing were investigated through a field measurement campaign and statistical analysis. The results showed that the increase of man-made surfaces due to urbanization has a significant positive effect on the magnitude of intra-urban air temperature variation, however, the effect varied among different times and seasons. Moreover, the influence of man-made surfaces on the magnitude of the intra-urban heat island effect increased with spatial extent. The different land cover types would have different effect on air temperature, but also indicated that the relative impacts of land cover on air temperature differed with time. Among the five land cover types, the PerBA was the mostly important factor increasing local air temperature, while the PerTC was the mostly important factor decreasing local air temperature. In addition, different land cover types had very different spatial extent dependence: with increasing spatial scale (the buffer zone size from 20 to 300 in radius), the correlation coefficient of different land cover types varied differently, and more interestingly, their relative impacts also varied among different times and seasons. In general, the moderating effects were significantly stronger at night than at noon, although they varied slightly with the seasons. Stepwise regression analysis indicated that ∼80% of the variation in temperatures can best be explained by the combination of the PerBA and PerPA at the scale of 200 m on winter night. In summer, ∼37% of the variation in temperature can best be explained by the PerTC at the scale of 20 m at noon, while ∼87% of the variation in temperature can best be explained by the PerBA and PerTC at the scale of 200 m at night. These findings provide valuable insights for the analysis of the local air temperature variation and will help to improve the data assimilated in urban climate models and surface energy balance applications. In addition, predictive models can be used to determine the air temperature variation that might result from land cover conversions due to urban expansion. Urban plan and landscape design professionals could also easily apply predictive models to provide guidance in their planning and design process for a comfortable thermal environment.

This work represents the first step in understanding the quantitative relationship between air temperature and land cover composition (a two-dimensional urban surface) at local scale. The study has also proved the existence of air temperature patterns that might be related to the urban canyon geometry (a three-dimensional urban structure). As Henry and Dicks [43] said that urban heat island, especially those that develop in the daytime, are perhaps most importantly determined by the urban canopy structure, and that the thermal properties of land cover are of secondary importance. Therefore, to explain the air temperature difference more precisely, both of land cover composition and structure need to be taken into account in future study. Furthermore, this research was conducted only for one metropolitan area, and under ‘ideal’ weather conditions (i.e. clear and windless). Whether this conclusion can be applied to different metropolitan areas and other climatic conditions should be further explored.
